# Osteoarthritis and telomere shortening

**DOI:** 10.1007/s13353-014-0251-8

**Published:** 2014-11-04

**Authors:** Lukasz Kuszel, Tomasz Trzeciak, Magdalena Richter, Malwina Czarny-Ratajczak

**Affiliations:** 1 Department of Medical Genetics, Poznan University of Medical Sciences, Rokietnicka 8, 60-806 Poznan, Poland; 2 Department of Orthopedics and Traumatology, Poznan University of Medical Sciences, 28 Czerwca 1956r. 135/147, 61-545 Poznan, Poland; 3 Department of Medicine, Tulane Center for Aging, School of Medicine, Tulane University, 1430 Tulane Ave., New Orleans, LA 70112 USA

**Keywords:** Osteoarthritis, Telomere length, Telomere shortening, Aging cartilage

## Abstract

Osteoarthritis is the most common disease of joints caused by degradation of articular cartilage and subchondral bone. It is classified as primary form with unknown cause and as secondary form with known etiology. Genetic and epigenetic factors interact with environmental factors and contribute to the development of primary osteoarthritis. Thus far, many polymorphisms associated with osteoarthritis have been identified and recent studies also indicate the involvement of epigenetic factors (e.g., telomere shortening) in the initiation of this disorder. Accelerated shortening of telomeres was detected in osteoarthritis and other age-related diseases. Studies revealed that telomere length is severely reduced in blood leukocytes and chondrocytes of patients with osteoarthritis, and this may contribute to the initiation and development of osteoarthritis, whose major cause is still unknown.

## Osteoarthritis

Osteoarthritis is characterized by the degradation of articular cartilage and subchondral bone, which results from an imbalance between cartilage degeneration and regeneration processes. Osteoarthritis is classified as primary (idiopathic) of unknown cause and secondary of known etiology. The current state of knowledge leads to the conclusion that, in primary osteoarthritis, genetic (Panoutsopoulou and Zeggini 2013) and epigenetic factors (Yamasaki et al. 2009; Li et al. 2012a, b) interact with environmental factors and contribute to osteoarthritis development and its further progression.

The most common causes of the secondary form are joint inflammation, obesity, post-traumatic changes, congenital deformation, joint dysplasias, diseases of the joints in childhood, metabolic disorders, hormonal imbalance, abnormal joint development, calcium crystal deposition, neuropathy, acromegaly, hemophilia, and a predisposition to systemic diseases (Rosenthal 2006; Buckwalter and Martin 2006). Osteoarthritis most frequently affects the hips, knees, spine, and joints of the hand (Dieppe and Lohmander 2005). The symptoms of the disease increase with age, and include mainly joint pain, contractures of the surrounding muscles, and gradual reduction in the range of motion. Radiological features of osteoarthritis are joint space narrowing, subchondral bone sclerosis, osteophytes, or bone cysts. Osteoarthritis is one of the diseases causing the most serious disabilities worldwide and generates enormous costs both directly (e.g., medical and non-medical costs) and indirectly (e.g., reduction in labor productivity) (March and Bachmeier 1997). The results of the Global Burden of Disease Study 2010 published in The Lancet (2012) show that 250,785,000 people are affected by knee osteoarthritis, which accounts for 83 % of the total osteoarthritis cases (Vos et al. 2012). Treatment of patients affected by osteoarthritis depends on the stage of diagnosis and is highly individualized. In mild osteoarthritis, the first approach is non-pharmacological treatment (e.g., education, exercise, diet, and physiotherapy) or intra-articular steroids or hyaluronic acid injections. More severe osteoarthritis may be treated pharmacologically with non-steroidal anti-inflammatory drugs (NSAIDs) or opioids. At the late stage of osteoarthritis, surgery involving the osteotomy or total joint replacement is the most common approach (Hunter and Felson 2006). Since osteoarthritis is considered a growing problem, particularly in the aging populations, many new treatments are currently being tested, including a biological therapy based on platelet-rich plasma, intra-articular cell therapy, or nanotechnology (Anitua et al. 2013; Peeters et al. 2013; Gu et al. 2013).

## Aging cartilage

In healthy cartilage, under normal conditions, cell divisions are practically minimal and aging is manifested as a slight decrease in the number of chondrocytes and thinning of the cartilage layers (Aurich et al. 2002; Horton et al. 2006; Li et al. 2013). Hudelmaier et al. (2001) conducted a study to assess the changes in the morphology of cartilage in the aging process. For the evaluation of the cartilage, they used magnetic resonance imaging, which revealed that cartilage in elderly patients, not affected by diseases and injuries within joints, is thinner than in young patients. They also showed that the reduction in cartilage thickness depends on gender and joint compartment. Changes that occur with age in cartilage predispose to joint injuries (Ding et al. 2005). Aurich et al. (2002) compared the cartilage for the content of main components of an extracellular matrix in young and aged donors. There were no significant differences in the content of type II collagen, glycosaminoglycans, and aggrecans between the two studied groups. In contrast, chondrocytes in the osteoarthritic cartilage lose their ability to synthesize matrix components and begin to synthesize proteins, which contribute to degradation of the extracellular matrix (Loeser 2009). Del Carlo and Loeser (2003) compared a response to oxidative stress of chondrocytes isolated from elderly and young individuals. The results indicated that chondrocytes isolated from the cartilage of the elderly are more susceptible to oxidative stress and, consequently, to the reactive oxygen species-associated cell death.

## Telomeres

Recent studies show that telomere shortening may became a significant epigenetic factor contributing to osteoarthritis. We analyzed the current literature about telomeres and reported telomere shortening in the blood and cartilage of patients with different forms of osteoarthritis. Telomeres are located at the ends of chromosomes. Human cells consist of a repeated telomeric sequence (TTAGGG)_n_, which is 5–15-kb long. Telomeres are necessary to maintain chromosome stability and prevent chromosome end fusion (Meyne et al. 1989; Tamayo et al. 2010). After each replication cycle, they are shortened by 50–150 bp (Harley et al. 1990; Counter et al. 1992). Enzyme telomerase, which is a large ribonucleoprotein complex, is able to reconstruct lost telomeric repeats and is active in stem cells and germ cells (Gilley et al. 2008). The telomerase complex is comprised of reverse transcriptase (TERT), RNA template for the synthesis of telomeric repeats, and the dyskerin protein complex (Chen et al. 2000; Bailey and Murnane 2006; Podlevsky et al. 2008). In most somatic cells of the human body, the lack of telomerase activity leads to the telomeres shortening, which is closely associated with the aging process and development of age-related diseases (Gilley et al. 2008). Besides telomerase, there are many important factors that play a role in the protection of chromosome ends. In human cells, there is a shelterin complex, consisting of six proteins. This complex includes telomeric repeat binding factors (TRF1 and TRF2), POT1 protein, and three linker proteins that allow the formation of a protein complex (TIN2, TPP1, and Rap1) (de Lange 2005; Martinez et al. 2010; Lu et al. 2013). TRFs function as an inhibitor of telomere elongation; POT1 protein binds to TTAGGG repeats, controls telomeres elongation by telomerase, and protects their ends. TIN2 protein is a central part of the shelterin complex and interacts with TPP1, TRF1, and TRF2. TPP1 protein interacts with the telomerase and directs it to the telomeres; Rap1 acts as a regulator of telomere function and of transcription (de Lange 2005; Martinez et al. 2010; Lu et al. 2013). Heterochromatin protein 1γ (HP1γ) binds to TIN2 protein and is involved in maintaining telomere cohesion (Canudas et al. 2011). The shelterin complex mediates the formation of telomere loops (T-loop) stabilized by smaller D-loops and, together, protect the telomere ends from fusing (Kong et al. 2013). SIRT6 protein plays a very important role in telomere protection and genome stability (Sharma et al. 2013). These investigators also reported that, in an *SIRT6* mouse knockout model, the aging process was very rapid. In addition, studies in cancer cells have revealed the involvement of the histone deacetylase 5 (HDAC5) in maintaining telomere length. Novo et al. (2013) reported significant telomere shortening in osteosarcoma and fibrosarcoma cells with no HDAC5 activity. In fibrosarcoma cells, they also observed a higher recombination potential of telomeres. These studies also demonstrated a higher sensitivity to chemotherapy in cancer cells lacking HDAC5 activity (Novo et al. 2013). The telomeric sequences form G4 structures (G-quadruplex) that may cause delay of the replication process and genomic instability (Lin et al. 2013). These structures are recognized and cut by DNA2 helicase/nuclease and results in telomere stabilization (Lin et al. 2013). Some studies indicate an important role of flap endonuclease 1 (FEN1) in maintaining genome stability through its participation in the replication process. Telomeres in cells lacking FEN1 activity show instability and dysfunction (Saharia et al. 2008, 2010).

Other proteins interacting with telomeres include tankyrase 1 and tankyrase 2, poly(ADP-ribose) polymerase 1 and 2 (PARP1/2), DNA-PK (DNA-dependent protein kinase), Ku70/80, MRN complex, ATM (ataxia telangiectasia mutated), MRE11, WRN (Werner’s syndrome protein), BLM (Bloom’s syndrome protein), DNA repair protein RAD51D, and others (Bailey and Murnane 2006; Verdun and Karlseder 2007).

With each replication cycle, telomeres gradually lose 50 to 150 bp, which is a natural process related to the telomere’s structure and DNA replication mechanism (Harley et al. 1990; Counter et al. 1992). Telomere shortening may be induced and accelerated by oxidative stress and DNA damage. Reaching the critical threshold of telomeres (Hayflick limit) leads to cellular senescence and, eventually, cell death (Gilley et al. 2008). Telomere dysfunction and shortening are linked to mitochondrial biology through the activation of P53, which affects the functioning of PGC-1α and PGC-1β, resulting in a decrease of mitochondrial mass and energy production (Sahin et al. 2011).

## Techniques utilized in telomere length measurements

Techniques used in the measurement of telomeres can be divided into two groups. The first group consists of molecular techniques and include techniques such as TRF (terminal restriction fragmentation), qPCR (quantitative polymerase chain reaction), MMqPCR (monochrome multiplex quantitative PCR), aTLqPCR (absolute telomere length quantitation), and STELA (single telomere length analysis). The second group consists of cytogenetic techniques based on fluorescence in situ hybridization (FISH), such as Q-FISH, PRINS, Flow-FISH, and HT Q-FISH. Various techniques and their advantages and disadvantages have been widely described by Montpetit et al. (2014). Briefly, most of the cytogenetic techniques are suitable for chromosome-specific analysis in small groups of individuals; they require more time compared to qPCR-based methods and more sophisticated equipment, like fluorescent or confocal microscopes. The molecular method TRF is considered the gold standard in the analysis of the mean telomere length; however, it is not designed for large groups of individuals. For such groups, more efficient are qPCR-based methods, such as qPCR, MMqPCR, and aTLqPCR. STELA allows for the measurement of critically short telomeres in a small set of chromosomes (Xp, Xq, 2p, 11q, 12q, and 17p). The Universal STELA method was developed to identify any short telomeres in the analyzed material.

## Telomere length and osteoarthritis

**Fig. 1:**
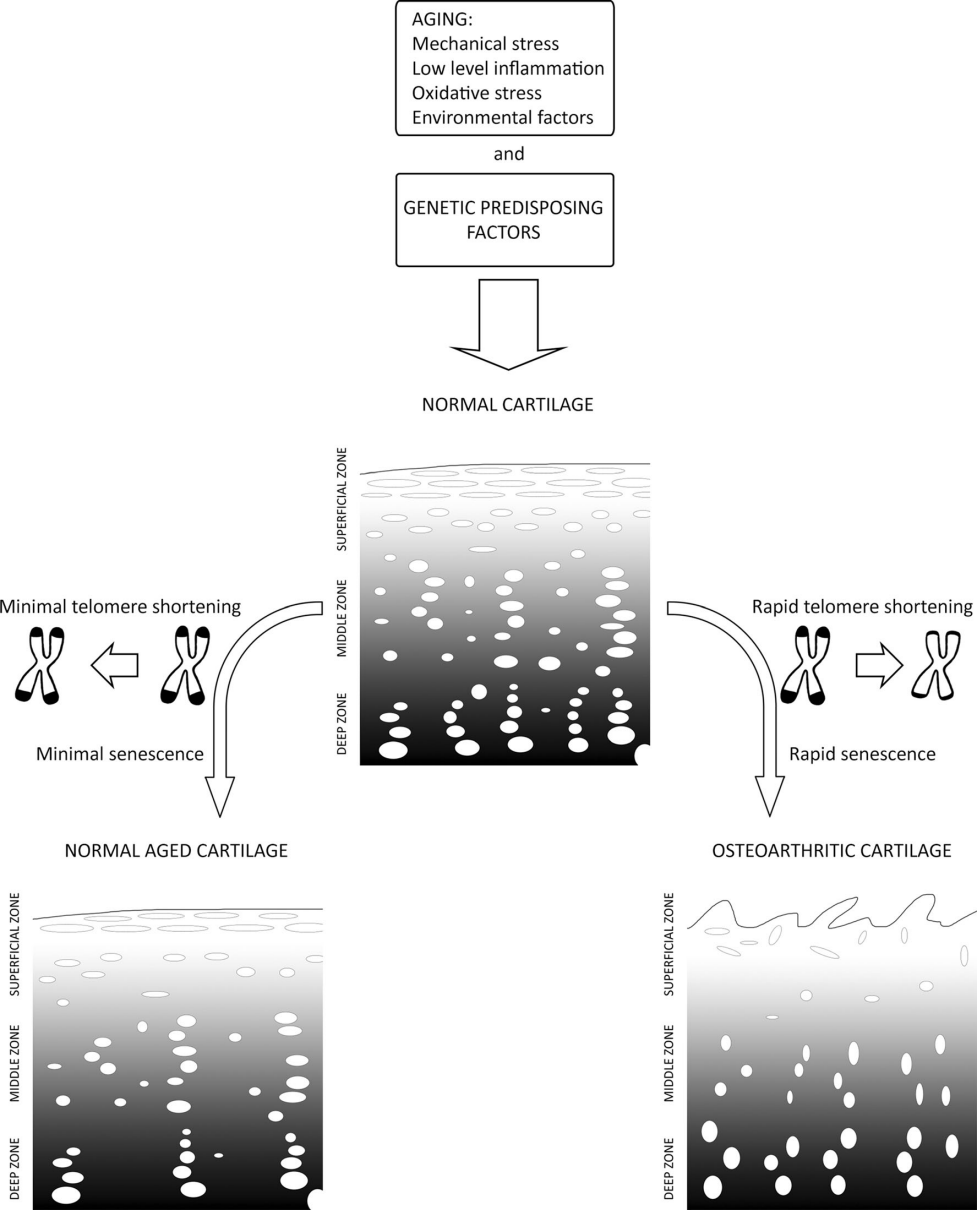
Aging and genetic factors contribute to the development of osteoarthritis, which is characterized by accelerated senescence, telomere shortening, changes in the structure of cartilage, and function of chondrocytes

Telomere shortening is involved in the pathogenesis of age-related diseases, among which osteoarthritis is one of the most common. Martin and Buckwalter (2001) conducted studies on a group of 27 patients, who were aged 1–87 years, and showed that age-related changes in human cartilage chondrocytes may lead to cartilage erosion and osteoarthritis. They also demonstrated that chondrocytes are devoid of telomerase activity (Martin and Buckwalter 2001). To assess the degree of telomere erosion, they utilized Southern blot analysis, by which they could estimate the mean terminal restriction fragment length (MTL). The results showed a significant difference in the MTL value between the young patients (11.8 kbp for a 13-year-old individual) and the old patients (8.7 kbp for an 87-year-old individual) (Martin and Buckwalter 2001). Price et al. (2002) used the same method to estimate telomere length in osteoarthritis patients and reported that chondrocytes obtained from the affected sites of osteoarthritis cartilage have shorter telomeres compared to unaffected chondrocytes. This research was carried out on an age-homogeneous group of 15 patients with hip osteoarthritis and 30 patients with knee osteoarthritis; the control group consisted of 11 patients with no joint diseases. Zhai et al. (2006) measured the relative telomere length in leukocytes of 1,086 patients (160 patients with hand osteoarthritis and 926 patients without hand osteoarthritis), and found that the telomeres of affected patients were shorter by 178 bp compared to the unaffected group. These results may indicate that leukocyte telomere length is a biomarker of osteoarthritis (Zhai et al. 2006; Li et al. 2012a, b). Telomere shortening in leukocytes in the course of osteoarthritis is most likely associated with exposure to oxidative stress and inflammatory states ongoing within the affected joints (Fig. 1). These factors can accelerate DNA replication, causing telomere loss (Zhai et al. 2006). In vitro studies of human chondrocytes showed the effects of oxidative stress on cellular aging, telomere instability, and erosion (Martin et al. 2004; Yudoh et al. 2005). Tamayo et al. (2010) studied telomere length in peripheral blood leukocytes of patients with rheumatologic diseases and osteoarthritis. An average telomere length was measured via qPCR. The analyzed group consisted of 34 patients with osteoarthritis and 130 controls; however, there was no difference in the telomere length between these two groups (Tamayo et al. 2010). In 2011, Tamayo et al. compared the telomere length of human chondrocytes and peripheral blood leukocytes. In this study, they analyzed a group of 20 control subjects and 39 osteoarthritis patients, consisting of 25 patients with knee osteoarthritis and 14 with hip osteoarthritis (Tamayo et al. 2011). In osteoarthritis patients, telomeres were 1.6 times longer in chondrocytes compared to leukocytes. In the control subjects, telomeres in chondrocytes were even twice as long as telomeres from leukocytes. The percentage of numerical chromosomal aberrations in chondrocytes from osteoarthritis patients was 1.5 times higher compared to leukocytes and 1.7 times higher than in chondrocytes from unaffected controls. No difference was detected between the leukocytes and chondrocytes of controls. Increased numerical chromosomal aberrations in the chondrocytes and leukocytes of osteoarthritis patients indicate that cells show signs of genomic instability, which is not limited to the affected joint. Shorter telomeres in leukocytes may result most likely from frequent divisions of leukocytes compared to chondrocytes (Tamayo et al. 2011). Rose et al. (2012) conducted a study on osteoarthritis cartilage collected during joint replacement from femoral condyles and compared it with cartilage collected during autopsies. The senescent phenotype was observed in chondrocytes affected by osteoarthritis; however, significant telomere shortening was not detected. This study showed a chaotic gene expression pattern and a significant increase in DNA damage in osteoarthritis chondrocytes compared to unaffected chondrocytes. In 2012, Harbo et al. (2012) collected tibia plateaus from three postmenopausal women at the ages of 56, 62, and 67 years. The telomere length was analyzed in cells from two sites differing in distance from the central osteoarthritic lesion. Quantification of ultrashort telomeres was performed via Universal STELA, and the mean telomere length was estimated with Q-FISH (Harbo et al. 2012). This study revealed that the distance from the center of the osteoarthritic lesion site is associated with telomere length just as a level of senescence and a level of osteoarthritis progression. In 2013, Harbo et al. analyzed tissue from the femoral heads of 14 osteoarthritis patients at the age of 60–86 years and nine patients without osteoarthritis at the age of 62–86 years. They revealed a correlation between the presence of ultrashort telomeres and distance from the central weight-bearing area of the joint in affected and unaffected individuals. In addition, the mean telomere length correlated with distance from the central weight-bearing area, except in the most central zone, where cells with longer telomeres were detected. Immunohistochemical analysis revealed that these are progenitor-like cells, which are most likely recruited to the degradated area of the joint (Harbo et al. 2013). Sibille et al. (2012) compared two groups of 18 individuals; the first group was affected by chronic stress and chronic pain caused by knee osteoarthritis, and the other group was a control group. They measured the telomere length in peripheral blood leukocytes using the qPCR method, described by Cawthon (2002), and found that telomeres were significantly shorter in patients from the affected group compared to healthy controls (Sibille et al. 2012). Most of these studies were conducted on small groups of patients, but they make important contributions to the understanding of the cellular senescence that occurs in cartilage tissue.

## Telomere shortening in other disorders

Telomere length may be associated with other diseases, such as cardiovascular disease, dementia, diabetes, cognitive decline, dyskeratosis congenita, aplastic and Fanconi anemia, idiopathic pulmonary fibrosis, cancer, and more (Martin-Ruiz et al. 2006; Fitzpatrick et al. 2011; Nilsson et al. 2013; Kong et al. 2013). Numerous studies indicate a link between telomere length in peripheral blood leukocytes and diseases unrelated to the hematopoietic system. Yan et al. (2011) reported that short telomeres in leukocyte are associated with aortic dissection. They studied white blood cells’ telomeres in 72 patients with aortic dissection, compared them to the telomeres of 72 health controls with no vascular disease, and, using qPCR, found that telomeres in leukocytes of patients with aortic dissection were significantly shorter than in the control group (Yan et al. 2011). These results are consistent with earlier findings. Wilson et al. (2008) carried out research on a group of 20 patients with asymptomatic abdominal aortic aneurysm (AAA) and on a control group consisting of 12 cadaveric organ donors. In both groups, they analyzed telomere length in the aortic biopsy as well as in the leukocytes, and found that the content of leukocyte and aortic telomere DNA was significantly lower in the patients with AAA compared to the control group (Wilson et al. 2008). Many researchers observed telomere shortening in leukocytes in other cardiovascular diseases, such as atherosclerosis (Samani et al. 2001) or large artery stiffness and cardiovascular burden (Wang et al. 2011). Sampson et al. (2006) reported that monocyte telomeres obtained from the blood of diabetic patients were shorter compared to the telomeres of the control group. Leukocyte telomeres showed no significant differences between the two groups, which may indicate that monocyte precursor cells could be exposed to high oxidative stress (Sampson et al. 2006). Honig et al. (2012) showed that short leukocyte telomeres are associated with dementia and mortality; the studied group consisted of 1,983 patients aged 65 years or older. Leukocyte telomere shortening was also observed in chronic obstructive pulmonary disease (COPD), which is supposed to be an age-related disease (Savale et al. 2009). Savale et al. (2009) compared three groups of patients: the first group was affected by COPD, the second group consisted of smokers, and third group with no respiratory diseases. They found that the leukocyte telomeres of COPD patients were significantly shorter than in the other two groups (Savale et al. 2009). Leukocyte telomere shortening is also observed in viral infections, such as hepatitis C. Kitay-Cohen et al. (2008) compared groups of chronic hepatitis C virus (HCV)-infected patients with patients after remission and a healthy control group. They showed that patients with chronic HCV infection had shorter telomeres than the healthy group. These studies show that telomere length in peripheral blood leukocytes can be a prognostic factor for other tissues.

In 2007, Njajou et al. (2007) studied telomere length in a large Amish group including 356 men and 551 women. They found telomere length to be negatively correlated with age and positively correlated with lifespan; their analysis also showed no significant differences in telomere length between men and women (Njajou et al. 2007). A very important point in this study was to show that offspring telomere length is correlated with paternal telomere length (Njajou et al. 2007). Eisenberg et al. (2012) demonstrated that offspring telomere length is dependent on the paternal age. Telomerase, functioning in the sperm, lengthens telomeres and are inherited by the offspring (Eisenberg et al. 2012). The most recent studies show that lifestyle and diet may affect the telomere length. Ornish et al. (2013) conducted a long-term study on two groups of men, ten men in the “lifestyle intervention group” and 25 men in the control group. Patients from the first group underwent lifestyle changes involving diet and activity (Ornish et al. 2013). After five years, the telomere length was estimated using qPCR. These analyses showed significant telomere shortening in the control group and increase of telomere length in the “lifestyle intervention group” (Ornish et al. 2013).

## Conclusions

Telomere shortening is a natural process that occurs in somatic cells, but accelerated shortening is observed in many diseases. Osteoarthritis is a condition in which, in addition to age, genetic, and environmental factors, telomere shortening may play a significant role (Fig. 1). Current research shows that measurements of telomere length in chondrocytes may be a valuable marker in the evaluation of cellular aging and prediction of osteoarthritis progression.

It seems that the most appropriate approach to measure telomere shortening is based on the comparison of samples collected from the same tissue (e.g., cartilage collected from more and less severely affected parts of the joint). This approach eliminates errors due to variation in telomere length between different individuals at birth. Unfortunately, this error is not eliminated while comparing different groups of individuals (e.g., cartilage collected from patients with osteoarthritis and unaffected controls). The mean telomere length seems to be a stable indicator of telomere length; however, it does not provide information about critically short telomeres, which is obtained via measurements with the Universal single telomere length analysis (STELA) method.

Currently, the diagnosis of osteoarthritis is mainly based on radiographic studies, which detect late stages of this disorder. A better understanding of molecular mechanisms involved in the initiation and progression of osteoarthritis may show new directions in the early diagnosis and new treatment methods of this extremely common population disorder. The identification of novel early biomarkers for osteoarthritis may allow to slow down osteoarthritis progression and help predict outcomes of osteoarthritis in individual patients. Telomere length and telomere shortening in leukocytes and chondrocytes could become early biomarkers for osteoarthritis; however, to make final conclusions, the analysis of larger and more homogenous groups needs to be performed. Thus far, most of the telomere studies were conducted on small groups of patients, frequently with knee or hip osteoarthritis combined into one group. In addition, more studies should be performed in order to better understand the effect of telomere shortening on cartilage aging.
